# Kleine-Levine syndrome in an adolescent female and response to modafinil

**DOI:** 10.4103/0972-2327.78052

**Published:** 2011

**Authors:** Ashish Aggarwal, Amit Garg, R. C. Jiloha

**Affiliations:** Department of Psychiatry, Indira Gandhi Medical College, Shimla, Himachal Pradesh, India; 1 Department of Psychiatry, GB Pant Hospital, New Delhi, India

**Keywords:** Females, Kleine-Levin syndrome, Modafinil

## Abstract

Kleine-Levine Syndrome (KLS) is a disorder characterized by a triad of periodic hypersomnia, hyperphagia, and hypersexuality. KLS, although more common in young males, it has also been seen in females. Treatment options available for its management include mood stabilisers like lithium, stimulants like amphetamines, antidepressants and other options including electroconvulsive therapy. Modafinil is one of the new stimulant medications approved for narcolepsy. Herein, we report a young female with KLS and showing favorable response to modafinil. More data is required to establish the effectiveness of modafinil in this syndrome.

## Introduction

Kleine-Levin syndrome is described as a triad of recurrent hypersomnolence, hyperphagia, and hypersexuality.[1] However, an incomplete presentation characterized by hypersomnia (which is mandatory for clinical diagnosis), with or without hyperphagia, hypersexuality, cognitive disturbances, mood symptoms, compulsive behaviors, and perceptual abnormalities has also been described.[2] KLS, though more common in males, has been reported to occur in females too.[3] Treatment options for management of KLS have generally met with limited success. Lithium has been reported to prevent relapses.[4] Other medications that have been used include antidepressants, antipsychotics, benzodiazepines, amphetamines, and even electroconvulsive therapy. Herein, a case of young female with KLS who showed favorable response to modafinil is described.

## Case Report

A 22-year-old unmarried female, with an uneventful birth and developmental history, without past and family history of any psychiatric or neurological illness, presented with an episodic illness of excessive sleep for past the six and a half years. The first episode started after a viral fever at 15 years of age. After a week of subsidence of fever, the first episode started abruptly with complaints of increased sleep, confusion, dream like state and irritability with intermittent irrelevant talks. She started sleeping more than her normal sleep. She would sleep for 16–18 hours in a day and would wake up for eating and natural calls. Sometimes she had to be awakened to have her meals. While eating, she would often eat more than her usual self. She could be arousable without difficulty but would prefer to go right back to sleep, would be irritable, and would not like to talk to anyone. The patient also complained that the things and the persons around her did not appear real to her. She would touch things to establish the reality. Her self-care also decreased and she was forced to take bath and change clothes. She also became fearful and would ask her mother to sleep with her. This was not present in premorbid state. She stopped going to the school. There was no significant personal or past history. Her menstrual history revealed irregular cycles and oligomenorrhea but this episode was not related to her menstrual period and had started around 10 days after her last menstrual cycle. A diagnosis of “Post viral depression” was made and she was started on multivitamins. This episode lasted for 13 days and there was complete spontaneous recovery. The patient had complete memory for the episode. In the next episode, 8 months later, a diagnosis of “Recurrent Depressive Disorder” was made and was started on sertraline 100 mg/day. In the next 2 years patient had 3 more episodes (8 and 3 months apart) each lasting for approximately 25 days, which occurred despite patient taking sertraline. During the fifth episode she got admitted and a diagnosis of “Periodic Hypersomnia” was made and started on methylphenidate upto 35 mg/day. This episode lasted for 35 days. During OPD follow-up, fluoxetine was added upto 40 mg/day. She remained symptom free for the next 2 years and did not follow-up. In the next 1 year she had 3 similar episodes (7 and 2 months apart) each being precipitated by sleep deprivation and lasting for approximately 15–20 days. She again got admitted during eight episode and a diagnosis of “Recurrent Hypersomnia” was made and she was restarted on methylphenidate, which was gradually increased to 25 mg/day. Her gynecological referral and investigations including serum prolactin, LH, FSH, and thyroid function test were normal. Her routine investigations including CBC, fasting blood sugar, KFT, urine R/M, and LFT were normal. Her ultrasonogram abdomen, MRI of the brain, and EEG were normal. This episode aborted in 12 days and she was continued on the same treatment. During few episodes in the past, the patient would eat excessively and also would talk regarding sex and on one occasion, started fondling with the genitalia of her cousin who had come. Following this she was symptom-free for 21 months though she underwent the treatment for 1 year only. The patient presented to us on the third day of ninth episode with similar symptoms. Outside the hypersomnia periods, she was asymptomatic. Patient did not cooperate for polysomnographic test, and hence, it could not be done. A diagnosis of Kleine-Levin Syndrome was made and she was started on modafinil 100 mg per day, which was increased to 200 mg/day on third day. She showed improvement in her symptoms from the fourth day of start of treatment and was subsequently discharged. She has been symptom-free after a total period of 2 years of regular follow-up on 100 mg of modafinil.

## Discussion

In 1898, a syndrome characterized by periodic hypersomnia and morbid hunger was described, but it was not until 1925 that a complete clinical delineation was made by a German psychiatrist, Willi Kleine. He described his first report on 5 boys. Additional affected males were described by Max Levin in 1929, and later in 1936. Critchley and Hoffman, who coined the term KLS in 1942, were the first to suggest that the syndrome exclusively affects adolescent boys. Later on, females with KLS were also reported.[3]

The common knowledge that females are an exception to this condition can explain the delay in the diagnosis of girls presenting with features of KLS.

Besides this, the exact prevalence of this syndrome is unknown and it is considered to be a rare disorder.

KLS is divided into primary and secondary forms depending on the absence or presence of neurological symptoms prior to KLS onset that persisted between episodes.[1]

The diagnosis of Kleine-Levin syndrome is based on clinical features alone, as there are no specific laboratory tests that can help in establishing the diagnosis of Kleine-Levin syndrome.[5] Besides hypersomnia and impairment of cognitive functions in the form of abnormal speech, confusion, amnesia, derealization, hallucinations and delusions, other symptoms that have been reported include eating behavior disorders like megaphagia, craving for sweets, increased drinking, binge eating, decreased appetite and food utilization behavior, depression, irritability, hypersexuality, and compulsions to sing, write, and pace.[1,6] Significant differences observed between primary and secondary forms include late age at onset, more incapacitation, long duration of episodes, and more number of episodes in patient with secondary form of KLS. The common causes associated with secondary KLS include stroke, posttraumatic brain hematoma, genetic or developmental diseases, multiple sclerosis, hydrocephalus, paraneoplasia in the context of a carcinoma of the cervix utero, an autoimmune encephalitis or a severe infectious encephalitis.[1]

Our case had presented with episodic course and spontaneous remission of each episode and normalcy in between the episodes. Characteristic features of episodes were hypersomnia, eating excessively, disinhibited behavior, affective features like irritability, social withdrawal, lack of personal care, and cognitive disturbance. The case had the onset after high grade fever, and also had features of confusion, dream-like state and intermittent irrelevant talks, which are also the important features of Kleine-Levin Syndrome.

The most important differential diagnosis for recurrent hypersomnia is the menstrual related hypersomnia. However, our patient did not have the episodes in relation to menstrual cycle and the episodes lasted longer than a week, which is the usual duration of episodes in menstrual related hypersomnia. Other important differential diagnosis include narcolepsy with and without cataplexy, idiopathic hypersomnia with and without long sleep time, recurrent hypersomnia, behaviorally induced insufficient sleep syndrome, hypersomnia due to medical condition, hypersomnia due to drug or substance, hypersomnia not due to a substance or known physiologic condition, and also sleep-related disordered breathing and periodic leg movement disorders.[7] Proper history and investigations in the form of sleep studies are important for the diagnosis. Our patient did not have any features suggestive of these disorders.

Limited literature is available on the management of KLS. Lithium had been reported to be useful but is associated with long-term problems of regular serum monitoring, side effects in females, and problems with pregnancy. Another option is using stimulants like methylphenidate. With increasing awareness amongst patients and also difficulty in procuring methylphenidate, there is a need for consideration of other treatment options for patients with KLS. Our cases responded to modafinil and it might be a safer alternative, although more duration of follow-up is warranted. Previous report on use of modafinil has shown inconsistent results.[1,8]

The prognosis of this disorder has generally been reported to be good, but still it is important to manage it appropriately and early to prevent its various complications.

There have been reports of various investigations in patients with KLS, although none has been reported to be specific for its diagnosis. EEG studies have shown a nonspecific diffuse slowing of background EEG activity, such as the alpha frequency band being slowed toward 7–8 Hz, was observed. Less often, low-frequency high amplitude waves (delta or theta) occurred in isolation or in sequence, mainly in the bilateral temporal or temporofrontal areas. However, one fourth of the patients had a normal EEG during episodes.[1]

Polysomnography done during the episode reveals reduced sleep efficiencies, reduced relative amonts of stage REM and stage 3–4 NREM sleep and increased relative NREM stage 1 sleep and wake time after sleep onset.[9] Multiple sleep latency test reveals short sleep latencies with sleep onset rapid eye movement periods possible in one or more naps.[9]

Our report suggests that for treating KLS one might use modafinil for its treatment. More systemic research is warranted to study the long-term effects of this drug on the course of KLS.
